# Arteriovenous shunting in patients with colorectal liver metastases.

**DOI:** 10.1038/bjc.1991.109

**Published:** 1991-03

**Authors:** J. A. Goldberg, J. A. Thomson, G. McCurrach, J. H. Anderson, N. Willmott, R. G. Bessent, J. H. McKillop, C. S. McArdle

**Affiliations:** University Department of Surgery, Royal Infirmary, Glasgow, UK.

## Abstract

The outlook for patients with colorectal liver metastases is poor. Microspheres have been combined with cytotoxics and administered via the hepatic artery in an attempt to improve tumour drug exposure within the liver. However, it has been suggested that arteriovenous connections might occur in association with intrahepatic tumours causing loss of regional advantage, and that the administration of microspheres further exacerbates arteriovenous shunting. In seven patients with colorectal liver metastases, base-line shunting was measured using a tracer quantity of radio-labelled albumin microspheres. The shunted fraction of a 'therapeutic quantity' of microspheres was subsequently measured in the same group of patients using albumin microspheres carrying a different radio-label. Base-line shunt for 0.5 x 10(6) microspheres was found to be 2.2 +/- 1.8% (mean +/- s.d.); the percentage shunt of a therapeutic quantity (40-80 x 10(6)) of microspheres was 3.0 +/- 0.8%. We conclude that arteriovenous shunting in patients with colorectal liver metastases is minimal, and is not significantly increased by the administration of therapeutic quantity of microspheres during regional chemotherapy.


Br. J. Cancer (1991), 63, 466-468 © Macmillan Press Ltd., 1991

### Arteriovenous shunting in patients with colorectal liver metastases

J.A. Goldberg[1], J.A.K. Thomson[2], G. McCurrach[2], J.H. Anderson[1], N. Willmott[3], R.G. Bessent[2], J.H. McKillop[4] & C.S. McArdle[1]

[1]*University Department of Surgery, Royal Infirmary, Alexandra Parade, Glasgow G31 2ER;* [2]*West of Scotland Health Boards Department of Clinical Physics and Bio-Engineering, and Department of Nuclear Medicine, Royal Infirmary, Glasgow;* [3]*Department of Pharmacy, University of Strathclyde, Glasgow; and* [4]*University Department of Medicine, Royal Infirmary, Glasgow, UK.*

**Summary** The outlook for patients with colorectal liver metastases is poor. Microspheres have been combined with cytotoxics and administered via the hepatic artery in an attempt to improve tumour drug exposure within the liver. However, it has been suggested that arteriovenous connections might occur in association with intrahepatic tumours causing loss of regional advantage, and that the administration of microspheres further exacerbates arteriovenous shunting. In seven patients with colorectal liver metastases, base-line shunting was measured using a tracer quantity of radio-labelled albumin microspheres. The shunted fraction of a 'therapeutic quantity' of microspheres was subsequently measured in the same group of patients using albumin microspheres carrying a different radio-label. Base-line shunt for $0.5 \times 10^6$ microspheres was found to be $2.2 \pm 1.8\%$ (mean ± s.d.); the percentage shunt of a therapeutic quantity ($40-80 \times 10^6$) of microspheres was $3.0 \pm 0.8\%$. We conclude that arteriovenous shunting in patients with colorectal liver metastases is minimal, and is not significantly increased by the administration of therapeutic quantity of microspheres during regional chemotherapy.

Conventional treatment of colorectal liver metastases has yielded disappointing results, and attention has turned to hepatic arterial chemotherapy. The potential advantages of regional therapy over systemic drug administration are that high drug levels can be achieved in the tumour-bearing organ and that systemic drug concentrations fall when the drug is retained within the organ, thereby minimising toxicity.

Particle-bound regional chemotherapy has been used in an attempt to improve drug uptake by the target organ. There are two mechanisms of value. Firstly, cytotoxic drugs have been co-administered with biodegradable microspheres which temporarily slow hepatic arterial blood-flow in the tumour-bearing liver and increase uptake of drug by the cells (Dakhil *et al.*, 1982; Thom *et al.*, 1989). Secondly, anti-cancer agents, including Adriamycin, mitomycin C, 5 fluorouracil, cis-platin and cytocidal radio-nuclides have been loaded into particles which act as controlled release vehicles in the target tissue (McArdle *et al.*, 1988; Fujimoto *et al.*, 1985; Okamoto *et al.*, 1986; Herba *et al.*, 1988).

If, however, arteriovenous shunting were associated with liver metastases (Zeissman *et al.*, 1983), a proportion of arterially administered drug would bypass vascular beds in the tumour-bearing liver and be carried to the lungs. The effect would be to reduce tumour drug exposure, increase systemic side-effects and in the case of some particle-bound cytotoxics, increase pulmonary toxicity.

With both approaches for enhancing regional therapy, relatively large numbers of particles may be required, either because of the relatively low drug pay-loads associated with some cytotoxic-loaded microspheres, or in order to optimise the arrest of arterial blood-flow. Unfortunately, there have been reports that very high levels of shunting occur when large numbers of microspheres are injected into the hepatic artery (Starkhammar *et al.*, 1987).

The aim of this study is to establish the level of base-line shunting and the effect of a therapeutic quantity of microspheres on shunting.

Correspondence: J.A. Goldberg
Received 23 July 1990; and in revised form 5 November 1990

### Patients and methods

### *Microsphere preparation*

It was necessary to design a radio-pharmaceutical specifically for this study. The requirements were for sterile radio-active microspheres (diameter 20–40 μm) which were bio-compatible, could be imaged by gamma camera, made in batches containing between 40 and $80 \times 10^6$ particles, and from which the activity would not leach.

$^{131}$I-labelled albumin microspheres were made in the following way. Under sterile conditions, 4.5 mg of $^{131}$I-human serum albumin (Medgenix, Brussels) supplied at a concentration of 20 mg $ml^{-1}$ was mixed with a solution of 380 mg cold human serum albumin (Sigma Chemicals) dissolved in 800 μl water containing 2 mg sodium dodecylsulphate. This consituted the disperse phase of a water in oil emulsion which was prepared with a Silverson mixer. The droplets were stablised by the addition of 240 μl 15% glutaraldehyde solution and the resulting microspheres separated and made ready for *in vivo* use as described elsewhere (Willmott *et al.*, 1985). For each batch of particles, the 50% weight average fell within the range of 25–40 μm.

The radiopharmaceutical was stable in air. In phosphate buffered saline, more than 99.7% of activity remained particle-bound after 2 months. The preparation was also remarkably stable in plasma. To check the stability of the radiolabelled particles in plasma, 2 mg of microspheres were incubated in 0.5 ml of serum at 37°C and after 10 days, particles were separated from supernatant and radioactivity in both measured. The amount of $^{131}$I released was expressed as a percentage of total activity. Between 92 and 96% of the original activity remained microsphere-bound after 10 days (median of 95%, $n = 6$).

A further *in vitro* experiment was performed to measure the sensitivity of albumin microspheres to protease digestion. Microspheres were incubated in a 0.4% trypsin solution at 37°C and degradation and solubilisation of particles recorded. The break-down pattern of microspheres by protease had a lag-phase which preceded swelling of the particles and was followed by degradation which was complete after 4 h (Willmott *et al.*, 1989).

### *Patients*

Seven patients with advanced colorectal liver metastases and indwelling hepatic artery perfusion catheters were included in the study. Informed consent was obtained. The extent of disease was assessed by tin colloid scan (less than 25% of liver mass in one; between 25 and 50% in four; greater than 50% in two). Each patient underwent two studies:

(a) An assessment of base-line arteriovenous shunting to lung for tracer quantities of microspheres;
(b) Measurement of the shunted fraction of a therapeutic quantity of microspheres.

The base-line shunt measurement was performed by injecting approximately half a million freshly prepared technetium labelled albumin microspheres (particle diameter 20–40 μ, Sorin Biomedica) via an hepatic artery catheter using a glass syringe. The activity of this tracer dose of particles was approximately 80 MBq. The liver and lungs were imaged by placing the patient supine under the gamma camera (IGE 400A Tomographic gamma camera with low energy, parallel hole general purpose collimator).

For the assessment of shunting of a 'therapeutic quantity' of albumin microspheres, $^{131}$I albumin microspheres were used. All patients were commenced on potassium iodate 2 days prior to administration of microspheres in order to prevent uptake of released radioactive $^{131}$I by the thyroid gland. This was continued for 14 days after microsphere administration.

The patient was positioned supine on the couch of the gamma camera (IGE 400A Tomographic gamma camera with high energy, parallel hole collimator was used). For each study, 200 mg of freshly prepared $^{131}$I-Albumin microspheres (containing between 40 and 80 × $10^6$ particles and between 2.7 and 8.7 MBq of activity) were drawn into a glass syringe, infused as a bolus into the hepatic artery catheter and the syringe flushed with 10 ml of saline to maximise delivery.

Anterior and posterior images of both lungs and liver were acquired immediately following injection of microspheres. Scans were acquired for 5 min or 500,000 counts, and stored on a dedicated computer in a 128 × 128 format (MAPS 2000, Link Analytical Ltd).

For each study, regions of interest were drawn on the images of the lung-fields from both the anterior and posterior views (excluding the cardiac region). A background region was drawn just outside the patient image, adjacent to the left lung. The geometric mean of the net anterior and posterior count rates was then found. Regions of interest were also drawn around the liver on both anterior and posterior views and the geometric mean of the net count rate found. The arteriovenous shunt percentage was calculated using the following formula:

$$\frac{\text{Lung count rate} \times 100}{\text{Liver + Lung count rate}} \%$$

### Results

The results are summarised in Table I. The median percent shunt was 1.3% at base-line. The median shunt after the therapeutic bolus of microspheres was 2.8%. The difference was not significant in relation to the errors of measurement.

### Discussion

High levels of base-line shunting (up to 26%) have been reported in patients with metastatic liver disease, and much higher levels have been reported when large numbers of biodegradable microspheres are co-administered with chemotherapy (Starkhammar *et al.*, 1987; Ensminger *et al.*, 1985). The radioactive particles used to measure arteriovenous shunting in these studies were macro-aggregates of albumin, surface-labelled with technetium. Potential errors with this radio-pharmaceutical include leaching of activity, the wide range of particle size (especially the diameter of the smallest particles), and the affinity of albumin aggregates for administration equipment (Palmer, 1985). Indeed we have previously shown that base-line shunting is minimal when these sources of error are accounted for (Goldberg *et al.*, 1987).

**Table I** Relative lung uptake in seven patients with advanced liver metastases

| *Patient* | *Base-line shunt (%)* *5 × $10^5$ microspheres* | *% of therapeutic dose shunted* *4–8 × $10^7$ microspheres* |
|---|---|---|
| 1 | 1.2 | 2.4 |
| 2 | 6.0 | 3.8 |
| 3 | 1.4 | 3.6 |
| 4 | 1.3 | 2.6 |
| 5 | 1.1 | 2.8 |
| 6 | 3.1 | 4.1 |
| 7 | 1.2 | 1.8 |

In the present study, in an attempt to circumvent these problems, we have used commercially available surface radiolabelled albumin microspheres with careful attention to preparation and handling, and the use of glass syringes to reduce the amount of free pertechnetate. Consequently, we have found values of base-line arterio-venous shunting to be consistently low, with only an exceptional value as high as 6% and five of seven values less than 1.5% (Table I). to assess the likelihood that free pertechnetate was the cause of the 6% result, a background area over the sternum was defined which would be expected to contain a contribution from blood-pool activity not trapped within the lung. Repeating the shunt calculation with this background gave a lung shunt percentage of 3.2%.

Little is known about the effect on shunting of the hepatic arterial injection of large numbers of particles, but it is important to exclude the potential loss of regional selectivity during hepatic arterial therapy. This would not only have a bearing on tumour exposure to the drug, but also increase pulmonary or systemic toxicity, depending on the cytotoxic formulation.

In this study, shunting was assessed during administration of a large quantity of particles which would be comparable in number to that used in a therapeutic setting. The number used had been found to be near the limit of tolerance in pilot studies for co-administration chemotherapy and biodegradable microspheres (Goldberg *et al.*, 1988). The use of commercially available surface-labelled albumin microspheres was not feasible when measuring the shunt associated with large numbers of microspheres because of the number of aliquots of microspheres required for the injectate, and their poor stability in air. Our customised $^{131}$I-labelled albumin microspheres proved to be a superior radio-pharmaceutical because of the covalent binding of the isotope throughout the particle matrix, resulting in minimal leaching of free radio-activity into the circulation.

In this study, we have confirmed that low levels of shunting occur in patients with colorectal liver metastases and the level of shunting is not significantly increased when large numbers of microspheres are administered via the hepatic artery. Arteriovenous shunting is therefore unlikely to increase the morbidity associated with particle-based therapy for patients in colorectal liver metastases.

We are endebted to the Cancer Research Campaign for financial support. We would like to thank both nursing and technical staff, Department of Nuclear Medicine, for their cooperation. We are grateful to Mr Alan Law (Office International, Glasgow) for his assistance with computer equipment.

### References

DAKHIL, S., ENSMINGER, W.D., CHO, K., NIEDERHUBER, J. DOAN, K. & WHEELER, R. (1982). Improved regional selectivity of hepatic arterial BCNU with degradable microspheres. *Cancer*, **50**, 631.

ENSMINGER, W.D., GYVES, J.W., STETSON, P. & WALKER-ANDREWS, S. (1985). Phase I study of hepatic arterial degradable starch microspheres and mitomycin. *Cancer Res.*, **45**, 4464.

FUJIMOTO, S., MIYAZAKI, M., ENDOH, F. & 5 others (1985). Effects of intra-arterially infused biodegradable microspheres containing mitomycin C. *Cancer*, **55**, 522.

GOLDBERG, J.A., BRADNAM, M.S., KERR, D.J. & 5 others (1987). Arteriovenous shunting of microspheres in patients with colorectal liver metastases: errors in assessment due to free pertechnetate and the effect of angiotensin II. *Nucl. Med. Commun.*, **8**, 1033.

GOLDBERG, J.A., KERR, D.J., WILLMOTT, N., MCKILLOP, J.H. & MCARDLE, C.S. (1988). Pharmacokinetics and pharmacodynamics of locoregional 5 fluorouracil (5FU) in advanced colorectal liver metastases. *Br. J. Cancer*, **57**, 186.

HERBA, M.J., ILLESCAS, F., THIRLWELL, M.P. & 4 others (1988). Hepatic malignancies: improved treatment with intra-arterial Yttrium$^{90}$. *Radiology*, **169**, 311.

MCARDLE, C.S., LEWI, H., HANSELL, D., KERR, D.J., MCKILLOP, J.H. & WILLMOTT, N. (1988). Cytotoxic-loaded albumin microspheres: a novel approach to regional chemotherapy. *Br. J. Surg.*, **75**, 132.

OKAMOTO, Y., KONNO, A., TOGAWA, K., KATO, T., TAMAKAWA, Y. & AMANO, Y. (1986). Arterial chemoembolisation with cisplatin microcapsules. *Br. J. Cancer*, **53**, 369.

PALMER, A.M. (1985). The adsorption of $^{99}Tc^{m}$-MAA onto vials and syringes. *Nucl. Med. Commun.*, **6**, 550.

STARKHAMMAR, H., HAKANSSON, L., MORALES, O. & SVEDBERG, J. (1987). Effect of microspheres in intra-arterial chemotherapy. A study of arterio-venous shunting and passage of a labelled marker. *Med. Oncol. & Tumour Pharmacother.*, **4**, 87.

THOM, A.K., SIGURDSON, E.R., BITAR, M. & DALY, J.M. (1989). Regional hepatic arterial infusion of degradable starch microspheres increases fluorodeoxyuridine (FUdR) tumour uptake. *Surgery*, **105**, 383.

WILLMOTT, N., CUMMINGS, J., STUART, J.F.B. & FLORENCE, A.T. (1985). Adriamycin-loaded albumin microspheres: preparation, *in vivo* distribution and release in the rat. *Biopharmaceutics & Drug Disposition*, **6**, 91.

WILLMOTT, N., CHEN, Y., GOLDBERG, J.A., MCARDLE, C.S. & FLORENCE, A.T. (1989). Biodegradation rate of embolised protein microspheres in lung, liver and kidney of rats. *J. Pharm. Pharmacol.*, **41**, 433.

ZEISSMAN, H.A., THRALL, J.H., GYVES, J.W. & 4 others (1983). Quantitative hepatic arterial perfusion scintigraphy and starch microspheres in cancer therapy. *J. Nucl. Med.*, **24**, 871.


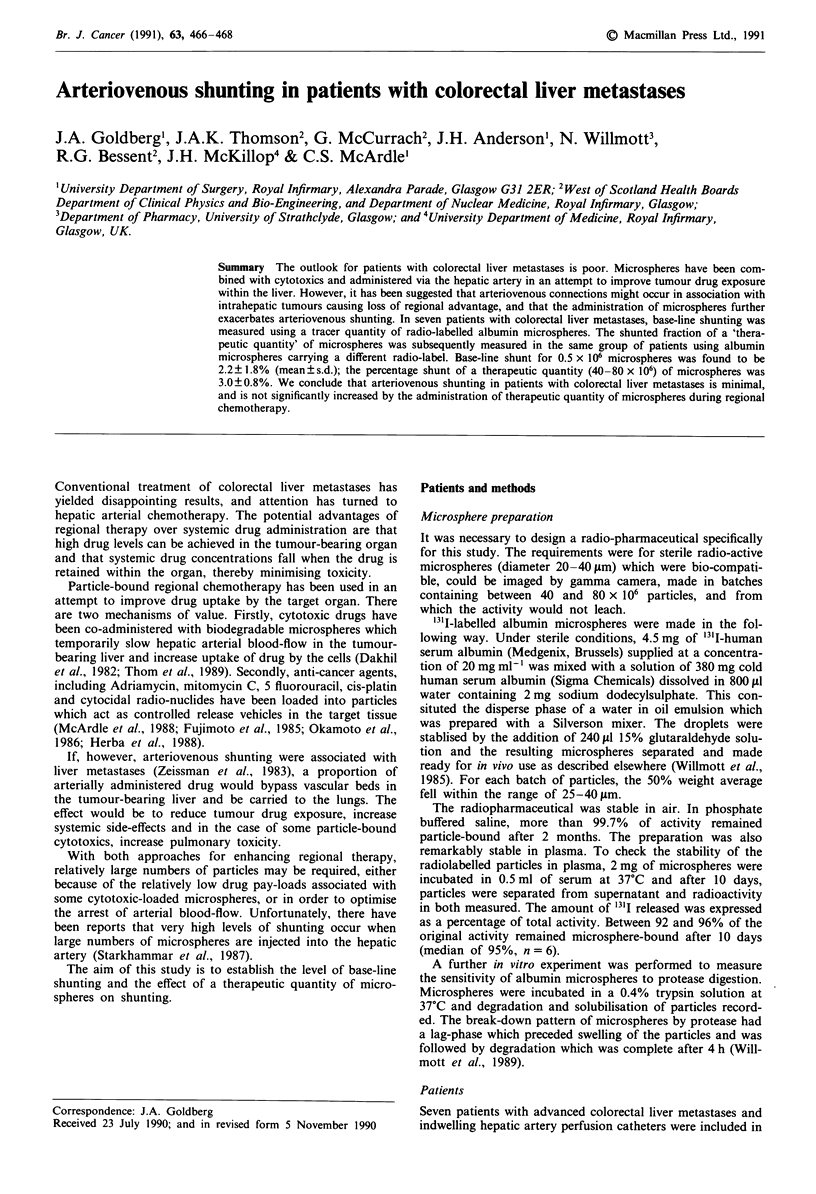

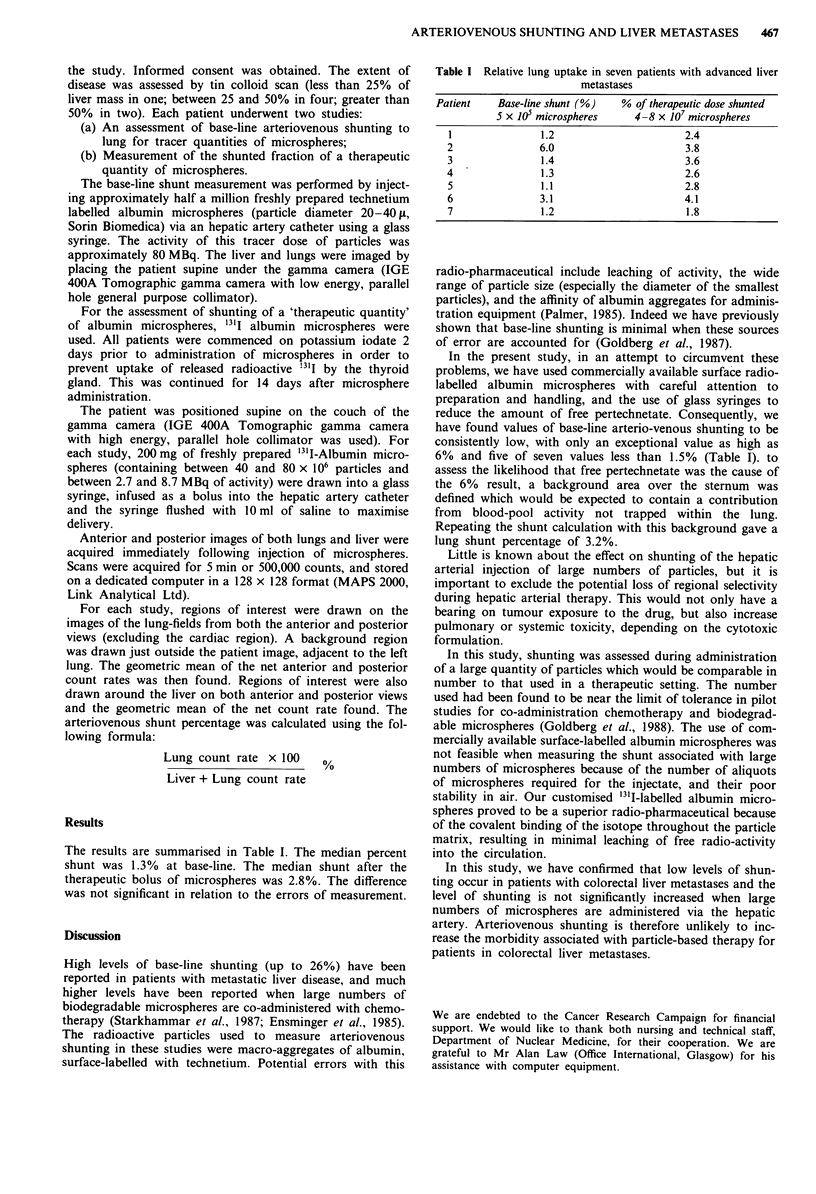

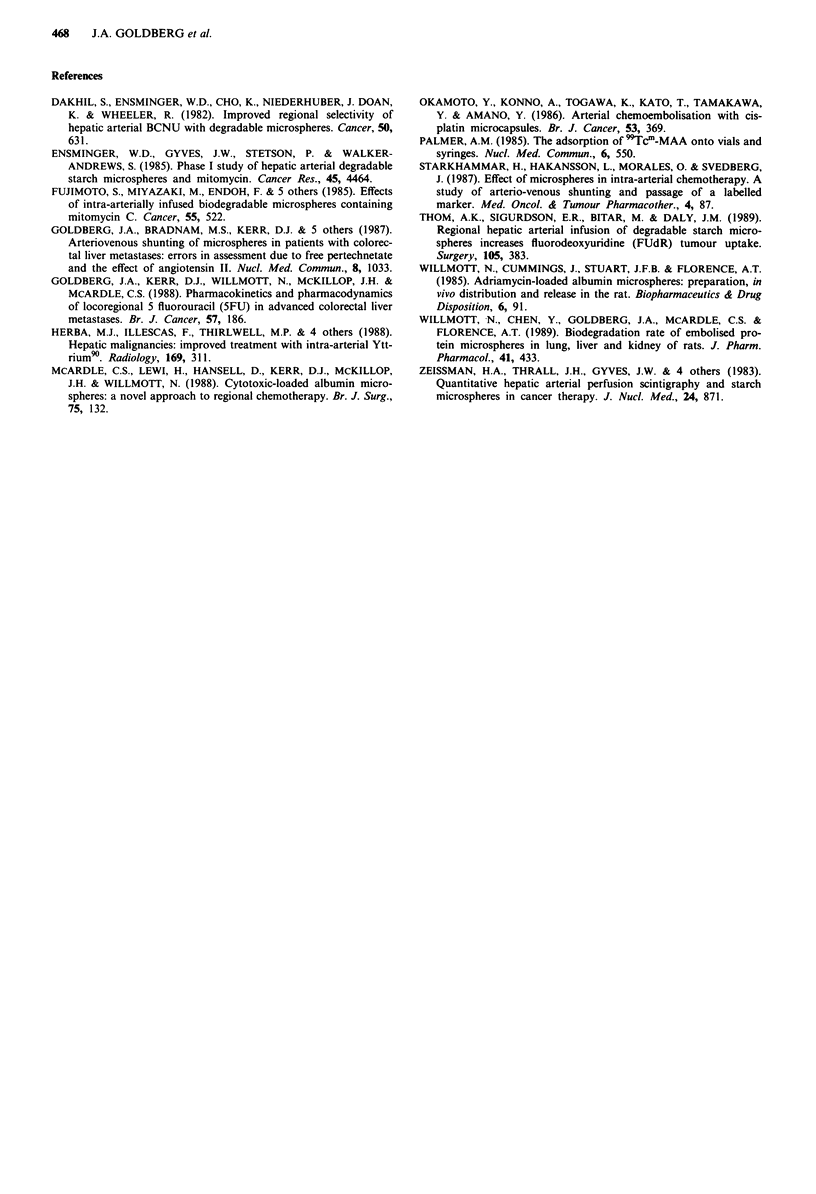


## References

[OCR_00290] Dakhil S., Ensminger W., Cho K., Niederhuber J., Doan K., Wheeler R. (1982). Improved regional selectivity of hepatic arterial BCNU with degradable microspheres.. Cancer.

[OCR_00298] Ensminger W. D., Gyves J. W., Stetson P., Walker-Andrews S. (1985). Phase I study of hepatic arterial degradable starch microspheres and mitomycin.. Cancer Res.

[OCR_00301] Fujimoto S., Miyazaki M., Endoh F., Takahashi O., Shrestha R. D., Okui K., Morimoto Y., Terao K. (1985). Effects of intra-arterially infused biodegradable microspheres containing mitomycin C.. Cancer.

[OCR_00306] Goldberg J. A., Bradnam M. S., Kerr D. J., Haughton D. M., McKillop J. H., Bessent R. G., Willmott N., McArdle C. S., George W. D. (1987). Arteriovenous shunting of microspheres in patients with colorectal liver metastases: errors in assessment due to free pertechnetate, and the effect of angiotensin II.. Nucl Med Commun.

[OCR_00311] Goldberg J. A., Kerr D. J., Willmott N., McKillop J. H., McArdle C. S. (1988). Pharmacokinetics and pharmacodynamics of locoregional 5 fluorouracil (5FU) in advanced colorectal liver metastases.. Br J Cancer.

[OCR_00317] Herba M. J., Illescas F. F., Thirlwell M. P., Boos G. J., Rosenthall L., Atri M., Bret P. M. (1988). Hepatic malignancies: improved treatment with intraarterial Y-90.. Radiology.

[OCR_00322] McArdle C. S., Lewi H., Hansell D., Kerr D. J., McKillop J., Willmott N. (1988). Cytotoxic-loaded albumin microspheres: a novel approach to regional chemotherapy.. Br J Surg.

[OCR_00328] Okamoto Y., Konno A., Togawa K., Kato T., Tamakawa Y., Amano Y. (1986). Arterial chemoembolization with cisplatin microcapsules.. Br J Cancer.

[OCR_00337] Starkhammar H., Håkansson L., Morales O., Svedberg J. (1987). Effect of microspheres in intra-arterial chemotherapy. A study of arterio-venous shunting and passage of a labelled marker.. Med Oncol Tumor Pharmacother.

[OCR_00343] Thom A. K., Sigurdson E. R., Bitar M., Daly J. M. (1989). Regional hepatic arterial infusion of degradable starch microspheres increases fluorodeoxyuridine (FUdR) tumor uptake.. Surgery.

[OCR_00355] Willmott N., Chen Y., Goldberg J., Mcardle C., Florence A. T. (1989). Biodegradation rate of embolized protein microspheres in lung, liver and kidney of rats.. J Pharm Pharmacol.

[OCR_00349] Willmott N., Cummings J., Stuart J. F., Florence A. T. (1985). Adriamycin-loaded albumin microspheres: preparation, in vivo distribution and release in the rat.. Biopharm Drug Dispos.

[OCR_00361] Ziessman H. A., Thrall J. H., Gyves J. W., Ensminger W. D., Niederhuber J. E., Tuscan M., Walker S. (1983). Quantitative hepatic arterial perfusion scintigraphy and starch microspheres in cancer chemotherapy.. J Nucl Med.

